# Inter-day reliability of heart rate complexity and variability metrics in healthy highly active younger and older adults

**DOI:** 10.1007/s00421-023-05373-3

**Published:** 2023-12-06

**Authors:** Christopher R. J. Fennell, Alexis R. Mauger, James G. Hopker

**Affiliations:** School of Sport and Exercise Sciences, University of Kent, Chipperfield Building, Canterbury, Kent, CT2 7PE UK

**Keywords:** Complexity, Ageing, Reproducibility, Heart rate

## Abstract

**Purpose:**

To investigate the inter-day reliability of time-domain, frequency-domain, and nonlinear HRV metrics in healthy highly active younger and older adults. The study also assessed the effect of age on the HRV metrics.

**Methods:**

Forty-four older adults (34 M, 10F; 59 ± 5 years; $\dot{V}{\text{O}}_{{{\text{2peak}}}}$  = 40.9 ± 7.6 ml kg^−1^ min^−1^) and twenty-two younger adults (16 M, 6F; 22 ± 4 years; $\dot{V}{\text{O}}_{{{\text{2peak}}}}$  = 47.2 ± 12.8 ml kg^−1^ min^−1^) attended the laboratory. Visit one assessed aerobic fitness through an exercise test. In visits two and three, participants completed a 30-min supine RR interval measurement to derive the HRV metrics.

**Results:**

The younger group (YG) and older group (OG) demonstrated poor to good day-to-day relative and absolute reliability for all HRV metrics (OG, ICCs = 0.33 to 0.69 and between day CVs = 3.8 to 29.2%; YG, ICCs = 0.37 to 0.93 and between day CVs = 3.5 to 36.5%). There was a significant reduction in ApEn (*P* < 0.001), SampEn (*P* = 0.031), RMSSD (*P* < 0.001), SDNN (*P* < 0.001), LF power (*P* < 0.001) and HF power (*P* < 0.001), HRV metrics with ageing. There was no significant effect of age the complexity metrics DFA α1 (*P* = 0.107), α2 (*P* = 0.147) and CI-8 (*P* = 0.493).

**Conclusion:**

HRV metrics are reproducible between days in both healthy highly active younger and older adults. There is a decline in linear and nonlinear HRV metrics with age, albeit there being no age-related change in the nonlinear metrics, DFA α1, α2 and CI-8.

## Introduction

Biological systems produce dynamic nonlinear outputs that are measurable across time, such as the variable fluctuations in the beat-to-beat (RRi) of the heart (Lipsitz and Goldberger 1992; Peng et al. 1995). The apparent “chaotic looking” behaviour of the fluctuations in an RR interval time series is accepted to contain meaningful structural richness; which can be assessed by using methods derived from nonlinear dynamics that can quantify the complexity (i.e., degree of self-similarity of fluctuations over multiple orders of temporal magnitude; Peng et al. 1995) and entropy (i.e., the regularity or randomness of the fluctuations; Richman and Moorman 2000) of the RR interval signal. While traditional linear time-domain methods provide a measure of variability between successive RR intervals, frequency-domain methods provide an estimation of the absolute or relative power of the RR interval signal (Shaffer and Ginsberg 2017).

Together the time-domain, frequency-domain, and nonlinear heart rate variability (HRV) metrics reflect the global functioning of the autonomic nervous system (ANS) through the interplay of sympathetic and parasympathetic activity at the sinus node (Task force 1996; Schwab et al. 2003). From a health-related and clinical perspective, a notable increase or decrease in heart rate complexity and variability away from an individual’s optimal range, may be indicative of an increased risk of sudden death, or adverse cardiac events such as arrythmias, myocardial infarcts, postural hypotension, and congestive heart failure (Kleiger et al. 1987; Goldberger et al. 1988; Lipsitz 1989; La Rovere et al. 1998; Stein et al. 2005). Moreover, research has shown a higher HRV to be positively associated with working memory (Mosley et al. 2018), cognitive performance (Hansen et al. 2004), emotional regulation (Williams et al. 2015) and incidence of depression (de la Torre-Lugue et al. 2016).

Research utilising a wide variety of HRV metrics has shown that during wakeful rest, both heart rate complexity (Kaplan et al. 1991; Iyengar et al. 1996; Pikkujamsa et al. 1999; Beckers et al. 2006; Voss et al. 2015) and variability (Jensen-Urstad et al. 1997; Umetani et al. 1998; Goff et al. 2010; Hernandez-Vicente et al. 2020) progressively decrease from early adulthood through to older age in healthy individuals. The World Health Organisation projects the number of people in the world over 60 years of age to increase from 1 billion (as of 2020) to 1.4 billion by 2030 and 2.1 billion by 2050 (data from who.int). Given the potentially negative physiological and psychological implications associated with a decrease in heart rate complexity and variability, it is pertinent there is continued research into the utility of HRV in older adults.

Previous research has assessed the intra and inter-day reliability of a few specific time-domain, frequency-domain (Al Haddad et al. 2011; Cipryan and Litschmannova 2013; Uhlig et al. 2020) and nonlinear HRV metrics (Maestri et al. 2007a). However, to the authors knowledge the inter-day reliability of the nonlinear HRV metrics has yet to be assessed in a homogenous group of healthy older adults. The current study therefore sought to extend upon the current literature investigating the reliability of HRV metrics, with the primary aim to provide new data on the day-to-day reliability of a range of HRV metrics in healthy active younger and older adults. The study also sought to assess the effect of age on HRV.

## Methods

### Participants

Sixty-six healthy individuals (50 male; 16 female) were recruited to participate in the study. Participants were divided into two age groups, the younger group (YG) were aged 18 to 30 years (*N* = 22; 16 M, 6F) and the older group (OG) were aged 50 to 70 years (*N* = 44; 34 M, 10F).

All participants were regular exercisers, having performed above the World Health Organisation guidelines (i.e., 2.5 to 5 h of moderate exercise per week; Bull et al. 2020) for ≥ 2 years. All participants were recruited to be closely matched for physical activity levels and exercise capacity. Participants were required to be non-obese, non-smokers, have no known or signs/symptoms of cardiovascular, neuromuscular, renal, or metabolic conditions and not be taking medications or dietary supplements that would affect cardiac function. The study was completed with full ethical approval of the University of Kent Research Ethics Committee (Proposal number: 21_2020_21), according to Declaration of Helsinki standards. All participants provided written informed consent prior to testing.

### Experimental design

Each participant completed three visits to the laboratory at the same time of day (± 1 h) between the hours of 8am and 4 pm (AM visits, YG *N* = 8 and OG *N* = 21; PM visits, YG *N* = 14 and OG *N* = 23). Visit one involved participant screening, laboratory familiarisation, and an incremental exercise test (IET) to determine aerobic fitness. At visits two and three, participants completed the 30-min supine resting RR interval measurement to derive the HRV metrics.

Visits were conducted on non-concurrent days (with a minimum gap of 2 full days and maximum gap of 5 days between visits) and participants were instructed to refrain from any exercise in the day prior to testing and intense exercise in the two days prior. Participants were instructed to arrive euhydrated and in a post-prandial state, having eaten at least 4-h prior to testing. Participants were told to not consume caffeine within 8-h and alcohol within 24-h of testing.

### Preliminary measurements and incremental exercise testing (visit one)

At visit one prior to exercise testing all participants provided written informed consent, completed a health questionnaire and the long form international physical activity questionnaire (Craig et al. 2003). Resting blood pressure, participant height, body mass and body composition were then measured, after which the participants completed a cycling IET to determine markers of aerobic fitness.

The IET protocol was performed on an electro-magnetically braked ergometer (Excalibur Sport, Lode BV, Groningen, The Netherlands). Participants completed a 10-min warm-up at 50 W, after which the required cycling power output increased by 25 W every minute (i.e., 1 W every 2.4 s) until they reached volitional exhaustion (operationally defined as a cadence of < 60 revolutions/min for > 5 s, despite strong verbal encouragement).

During the IET, respiratory gas exchange data were assessed using online breath-by-breath gas analysis (Metalyzer 3B; CORTEX Biophysik GmbH, Leipzig, Germany). Prior to all testing the gas analyser was calibrated according to the manufacturer recommendations using with ambient air and known concentrations of oxygen and carbon dioxide. The bidirectional turbine (flow meter) was calibrated with a 3-L calibration syringe.

The participant’s peak oxygen uptake ($\dot{V}{\text{O}}_{{{\text{2peak}}}}$) was assessed as the highest oxygen uptake that was attained during a 1-min period in the test. Participants gas exchange threshold was determined as the breakpoint in carbon dioxide production and oxygen consumption (i.e., the point at which the carbon dioxide production begins to increase out of proportion to the oxygen consumption). This breakpoint also coincided with the increase in both ventilatory equivalent of oxygen ($\dot{V}{\text{E}}/\dot{V}{\text{O}}_{{2}}$) and end-tidal pressure of oxygen with no concomitant increase in ventilatory equivalent of carbon dioxide ($\dot{V}{\text{E}}/\dot{V}{\text{CO}}_{{2}}$; Beaver and Wasserman 1986; Pallares et al. 2016). The respiratory compensation point was determined as an increase in both the $\dot{V}{\text{E}}/\dot{V}{\text{O}}_{{2}}$ and $\dot{V}{\text{E}}/\dot{V}{\text{CO}}_{{2}}$and a decrease in partial pressure of end-tidal carbon dioxide (Whipp et al. 1989; Lucia et al. 1999).

### Measurement of RR intervals (visits two and three)

For collection of RR intervals participants were in a supine resting position, in a temperature-controlled room set at 20 C. The room was kept dark and quiet, and participants were instructed not to verbalise throughout the measurement and breathe freely at their normal resting rate. Before the 30-min RR interval measurement commenced, an initial 20-min supine rest period was carried out to ensure participants were at complete rest and their heart rates were stable.

To collect the RR intervals participants wore a Polar H10 heart rate monitor with a Pro Strap (Polar Electro Oy, Kempele, Finland), which has been shown to provide strong agreement and comparable RR interval signal quality to conventional ECG devices (Gilgen-Ammann et al. 2019; Schaffarczyk et al. 2022). The elastic electrodes of the Pro Strap were moistened, and the strap lengthened to fit around the participant’s chest circumference as described by the manufacturer. The RR intervals were acquired at 1000 Hz via the Elite HRV application (Elite HRV, Asheville, NC, USA) on a mobile device positioned directly next to the participant. The RR intervals were then exported as a text file for processing and analysis offline in MATLAB.

### RR interval data pre-processing

All RR interval time series were pre-processed to exclude artifacts and outliers. RR intervals less than 0.2 s and greater than 2.0 s were removed. Secondly, RR intervals that differed from the mean of the surrounding 40 RR intervals by more than 20% were excluded.

The number of RR interval artifacts and outliers from all RR interval time series on Day 1 were: YG, 19.6 ± 20.5 RR intervals or 1.12 ± 1.24% (range 0.05 to 4.33%) of total RR intervals and OG, 7.5 ± 10.6 RR intervals or 0.46 ± 0.64% (range 0.00 to 2.65%) of total RR intervals and Day 2: YG, 16.3 ± 15.9 RR intervals or 0.94 ± 0.94% (range 0.00 to 3.03%) of total RR intervals and OG, 6.7 ± 12.1 RR intervals or 0.42 ± 0.76% (range 0.00 to 4.10%) of total RR intervals.

### Heart rate complexity—nonlinear metric analysis

#### Approximate and sample entropy

Approximate entropy (ApEn; Pincus 1991) and sample entropy (SampEn; Richman and Moorman 2000) quantify the conditional probability that a template length of *m* and *m* + 1 data points is repeated during the time series within a tolerance of *r* (set at a % of the time series SD)*.* SampEn differs from ApEn, as it avoids counting self-matches by taking the logarithm after averaging, thus reducing the inherent bias existing within the ApEn calculation.

In the current study template length was set at *m* = 2 and tolerance *r* = 0.2 of the SD of the RR interval time series, for both ApEn and SampEn analysis (Kaplan et al. 1991). ApEn was calculated as shown by Eq. (1) and SampEn by Eq. (2), where *N* is the number of data points in the time series, *m* is the length of the template, *Ai* is the number of matches of the *i*th template of length m + 1 data points, and *Bi* is the number of matches of the *i*th template of length *m* data points:

1 $$ApEn\left( {m,r,N} \right) = { }\frac{1}{{N - {\text{m}}}}\mathop \sum \limits_{i = 1}^{N - m} log\frac{{A_{i} }}{{B_{i} }}$$

2 $$SampEn\left( {m,r,N} \right) = - {\text{ log}}\left( {\frac{{\sum \begin{array}{*{20}c} { N - m } \\ { i = 1 A_{i} } \\ \end{array} }}{{\sum \begin{array}{*{20}c} {N - m} \\ {i = 1} \\ \end{array} B_{i} }}} \right)$$

### Detrended fluctuation analysis

The detrended fluctuation analysis (DFA) algorithm was used, as outlined by Peng et al. (1994), to measure the fractal scaling of the RR interval time series. The DFA algorithm allows for the detection of long-range correlations embedded in seemingly non-stationary physiological time series data. The RR interval time series is first integrated, using Eq. (3):

3 $$y(k) = \sum_{j=1}^{k}({RR}_{j }- \overline{RR }), k = 1, ...,N$$

The integrated time series are then divided into boxes of equal length, *n*. Within each box length *n*, a least squares line is fitted to the data, y_n_(k) denotes the trend in each box. The integrated time series y(k) is then detrended by subtracting the local trend, y_n_(k), within each box. The root-mean-square fluctuation of the integrated and detrended time series is calculated by Eq. (4):

4 $$F\left( n \right) = { }\sqrt {\begin{array}{*{20}c} \frac{1}{N} & {\mathop \sum \limits_{k = 1}^{N} \left[ {y\left( k \right) - y_{n } \left( k \right)]^{2} } \right.} \\ \end{array} }$$

The DFA computation (4) is repeated across all box sizes to provide a relationship between *F*(*n*), the average fluctuation as a function of box size, and the box size, *n*, the number of RR interval data points in a box. The slope of the double log plot, log *F*(*n*) vs log *n,* determines the scaling exponent α. DFA α was calculated with box sizes ranging from 4 to $\le$ 64 data points. DFA α1 was calculated over box sizes of 4 $\le$ *n* $\le$ 16 data points (i.e., scaling exponent calculated over short time scales) and DFA α2 was calculated over box sizes of 16 $\le$ *n* $\le$ 64 data points (i.e., scaling exponent calculated over long time scales), as used previously by Peng et al. (1995).

**Fig. 1:**
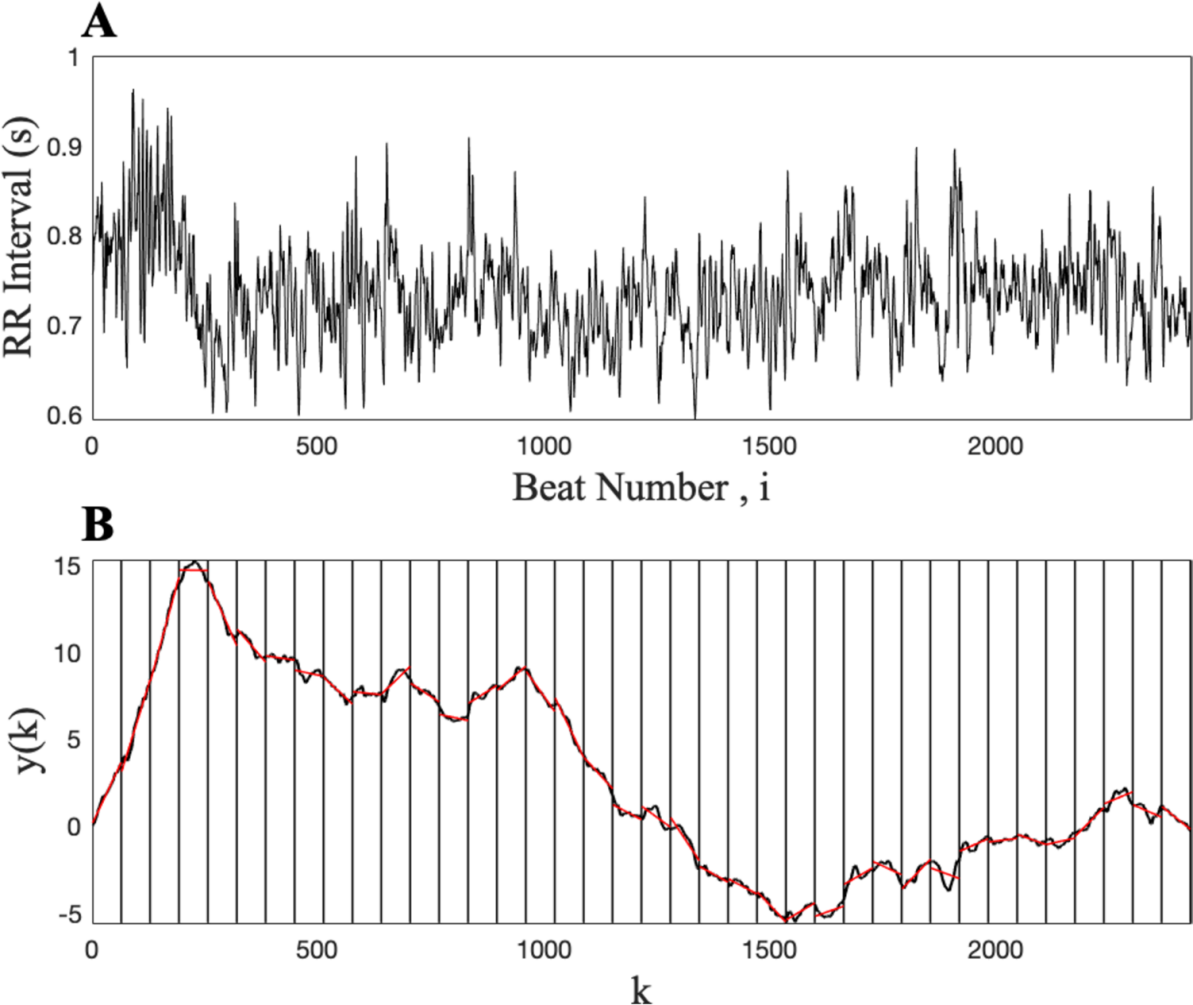
**A** Example raw RR interval time series; **B** the integrated RR interval time series, with the least-squares fit representing the “trend” in each box (red lines) and the vertical lines indicating the box size of *n* = 64 data points. The RR interval data presented produced a DFA α = 1.04 (DFA *α* calculated over box sizes 4 to $\le$ 64; data were from a younger male participant aged 18 years)

The DFA produces a scaling exponent *α*. An *α* = 0.5 indicates that the value of one RR interval is completely uncorrelated from any previous values (i.e., unpredictable white noise; indicative of a very rough time series). An *α* = 1.5 indicates Brown noise and a loss of long-range correlations (i.e., a smooth output with long term memory). While an *α* of 1.0 (i.e., 1/f or pink noise) is suggestive of a physiological output of high complexity*,* that is statistically self-similar with long range-correlations (Peng et al. 1995). Figure 1A presents an example raw RR interval time series and 1B presents the integrated time series with the least-squares fit “trend” line plotted for box sizes of 64 data points.

#### Multiscale entropy

Multiscale entropy (MSE) analysis was performed as outlined by Costa et al. (2002) providing a measure of complexity of time series over multiple scales. The MSE analysis overcomes limitations of SampEn and ApEn which only measure the regularity of time series data on one scale, and therefore do not capture the structural and dynamical behaviour of the time series.

From the one-dimensional discrete time series, {χ_1_,…, χ_I_,…, χ_*N*_}, a coarse-grained time series were constructed, {y^(τ)^}, determined by the scale factor, τ, according to Eq. (5):

5 $$y\begin{array}{*{20}c} {\left( \tau \right)} \\ j \\ \end{array} = \frac{1}{\tau } \mathop \sum \limits_{{i - \left( {j - 1} \right)\tau + 1}}^{j\tau } \chi_{i^{\prime}} 1 \le j \le N/\tau$$

At one scale, the time series {y^(1)^} is the original time series of sample length. The length of the coarse-grained time series is equal to the length of the original time series divided by the scale factor, τ. The SampEn for each coarse-grained time series is calculated and plotted against the scale factor, τ, producing a MSE curve. The SampEn of each coarse-grained time series was computed using Eq. (2) and a template length *m* = 2 and *r* = 0.2 of the SD of the RR interval time series. The area under the MSE curve were calculated from scales 1 to 8 using Eq. (6) and is defined as the complexity index (CI-8) with higher CI values indicating greater complexity of the physiological signal.

6 $${\text{CI }} = \mathop \sum \limits_{i = 1}^{\tau } SampEn\left( i \right)$$

#### Poincare plot SD2

Poincare plots of RR interval times series were produced by plotting each RR interval as a function of the previous RR interval (Woo et al. 1992). Poincare plots were then analysed with an ellipse fitting procedure to derive the metrics SD1 (the standard deviation of the points perpendicular to the line of identity) and SD2 (the standard deviation along the line of identity; Brennan et al. 2001). Only SD2 was reported as SD1 is identical to RMSSD (Shaffer and Ginsberg 2017).

### Heart rate variability—linear metric analysis

#### Time-domain metrics

The time-domain measures of heart rate variability quantify the amount of variability present within the RR interval time series.

The root mean square of successive differences between normal RR intervals (RMSSD) was calculated using Eq. (7):

7 $${\text{RMSSD}} = { }\sqrt {\begin{array}{*{20}c} {\frac{1}{N - 1}} & {\mathop \sum \limits_{n = 1}^{N - 1} (RR_{n + 1} - RR_{n } )^{2} } \\ \end{array} }$$

The standard deviation of normal RR intervals (SDNN) was calculated using equation (8):

8 $${\text{SDNN}} = { }\sqrt {\begin{array}{*{20}c} {\frac{1}{N - 1}} & {\mathop \sum \limits_{n = 1}^{N } (RR_{n} - \overline{RR} )^{2} } \\ \end{array} }$$

The RMSSD and SDNN metrics were reported in milliseconds and natural logarithm transformed values, LnRMSSD and LnSDNN.

#### Frequency-domain metrics

The frequency-domain measures of heart rate variability provide an estimate of spectral power in frequency bands. The power spectrum was estimated using a parametric autoregressive based model, with the absolute power in the low frequency power (LF) band (0.04–0.15 Hz) and high frequency power (HF) band (0.15–0.4 Hz) calculated, along with the LF/HF ratio. The absolute power in the LF and HF band is reported in ms^2^ and natural logarithm transformed values (Ln).

### Statistical analysis

Data are presented as individual values or mean ± SD (unless specified otherwise). Statistical analyses were conducted using IBM SPSS Statistics 29 (IBM, Armonk, New York, USA). Visual inspection of Q-Q plots and Shapiro–Wilk statistics were used to check whether data were normally distributed.

Day-to-day reliability of all heart rate complexity and variability metrics was assessed through a two-way random intraclass correlation coefficient (ICC2,1) for absolute agreement, standard error of measurement (SEM), minimal detectable change (MDC) and Bias (being mean difference between day 1 and day 2). Upper and lower 95% limits of agreement (LOA) were calculated as the mean of differences between days ± 1.96 × the standard deviation of the differences. Between day coefficient of variations (CVs) of all HRV metrics were calculated by dividing the SD of both days’ measurement by the mean of both days measurement and multiplying by one hundred. Between participant CVs for all HRV metrics were calculated by dividing the SD of all participant measurement by the mean of all participant measurement and multiplying by one hundred. Paired samples *t****-***tests were used to assess whether a significant difference in the complexity and variability metrics were present between days for each age group.

Based on the ICCs, relative reliability was defined as: poor = ICC < 0.5, moderate = ICC ≥ 0.5 to < 0.75, good = ICC ≥ 0.75 to < 0.90 and excellent = ICC ≥ 0.90 (Koo and Li 2016).

Hedges’ g effect sizes and the 95% confidence intervals were calculated to assess the differences between the two age groups (YG vs. OG) HRV metrics and interpreted as: 0.2 to 0.5 small effect, 0.5 to 0.8 medium effect, ≥ 0.8 large effect (Cohen 1992).

Multiple linear regressions were performed to estimate the effect of participant age, sex and $\dot{V}{\text{O}}_{{{\text{2peak}}}}$ on all heart rate complexity and variability metrics. Males were set as the baseline reference level; therefore, positive beta coefficients indicate that being female will likely result in a higher value.

The significance level was set at *P* < 0.05 in all cases.

## Results

### Participant characteristics and anthropometrics

Data from forty-four older adults (34 M; 10F) and twenty-two younger adults (16 M; 6F) were included in the analysis. Table 1 presents participant anthropometrics and IET data.

**Table 1:** Participant characteristics, anthropometrics and IET data (mean ± SD)

	OG	YG
*N*	44 (34 M; 10F)	22 (16 M; 6F)
Age (years)	58.6 ± 5.1	21.9 ± 3.7
Height (cm)	173.8 ± 8.6	177.3 ± 9.8
Mass (kg)	72.3 ± 12.1	74.1 ± 12.1
Fat mass (%)	22.0 ± 7.2	16.1 ± 9.1
Lean body mass (%)	78.0 ± 7.2	83.9 ± 9.1
Lean body mass (kg)	56.3 ± 10.1	61.9 ± 10.9
Lean body mass index (kg m^2^)	18.5 ± 2.1	19.3 ± 1.9
Systolic BP (mmHg)	130.6 ± 7.9	126.1 ± 6.0
Diastolic BP (mmHg)	80.3 ± 9.6	73.4 ± 7.8
Absolute $\dot{V}{\text{O}}_{{{\text{2peak}}}}$ (L min^−1^)	3.0 ± 0.8	3.5 ± 1.0
Relative $\dot{V}{\text{O}}_{{{\text{2peak}}}}$ (ml kg^−1^ min^−1^)	40.9 ± 7.6	47.2 ± 12.8
Power at $\dot{V}{\text{O}}_{{{\text{2peak}}}}$ (W)	277.2 ± 68.2	318.1 ± 94.4
Relative $\dot{V}{\text{O}}_{{2}}$ at GET (ml kg^−1^ min^−1^)	27.2 ± 6.7	31.5 ± 10.3
Power at GET (W)	162.1 ± 47.7	193.0 ± 71.6
Relative $\dot{V}{\text{O}}_{{2}}$ at RCP (ml kg^−1^ min^−1^)	34.3 ± 7.1	38.4 ± 10.9
Power at RCP (W)	215.3 ± 56.6	242.4 ± 80.3
Exercise time per week (hours)	9.9 ± 4.7	13.2 ± 4.8
MET hours per week	85.9 ± 49.4	104.1 ± 52.4

*OG* Older group; *YG* younger group; *BP* blood pressure; $\dot{V}{\text{O}}_{{{\text{2peak}}}}$ peak oxygen uptake; $\dot{V}{\text{O}}_{{2}}$ oxygen uptake; *GET* gas exchange threshold; *RCP* respiratory compensation point; *MET* metabolic equivalents

### Reliability of heart rate complexity and variability-based metrics

Based upon the ICCs the OG demonstrated poor reliability for the CI-8 and SD2 metric, moderate reliability for the RMSSD, SDNN, LnRMSSD, LnSDNN, LF(ms^2^), HF(ms^2^), LF(log), HF(log), ApEn, SampEn, DFA α, DFA α1 and DFA α2 metrics, and good reliability for the LF/HF metric (Table 2). By comparison, the YG demonstrated poor reliability for the ApEn, SampEn and SD2 metrics, moderate reliability for the LnSDNN, LF (ms^2^), LF(log), DFA α2 and CI-8 metrics, good reliability for the RMSSD, SDNN, LnRMSSD, HF(ms^2^), HF(log), LF/HF and DFA α metrics and excellent reliability for the DFA α1 metric (Table 3).

**Table 2:** Older group day-to-day reliability of RR interval complexity and variability metrics

	Between Day CV (%)	Between Participant CV (%)	ICC2,1	SEM	MDC	Bias	SD bias	Lower 95% LOA	Upper 95% LOA	*P*
HR (bpm)	4.36	11.64	0.79	2.89	8.00	– 0.20	4.08	– 8.21	7.80	0.74
RRi (s)	4.13	11.81	0.83	0.05	0.15	< 0.01	0.08	– 0.15	0.15	0.83
RMSSD (ms)	17.25	44.11	0.61	10.70	29.66	0.71	15.13	– 28.95	30.37	0.76
LnRMSSD	5.09	11.92	0.57	0.28	0.77	0.01	0.39	– 0.76	0.77	0.89
SDNN (ms)	14.8	28.0	0.62	10.06	27.88	– 4.09	14.23	– 31.97	23.79	0.06
LnSDNN	3.77	6.74	0.53	0.19	0.52	– 0.08	0.26	– 0.60	0.44	0.05
LF (ms^2^)	29.22	83.08	0.69	349.65	969.18	– 84.91	494.48	– 1054.09	884.27	0.28
HF (ms^2^)	28.91	87.28	0.65	239.02	662.54	30.81	338.03	– 631.72	693.35	0.53
LF (Ln)	4.87	10.86	0.69	0.39	1.07	– 0.16	0.55	– 1.24	0.91	0.06
HF (Ln)	5.80	14.73	0.62	0.53	1.46	– 0.03	0.74	– 1.49	1.42	0.79
LF/HF (ratio)	27.07	112.92	0.88	1.16	3.23	– 0.25	1.65	– 3.48	2.97	0.33
ApEn	2.95	6.45	0.60	0.06	0.17	– 0.02	0.09	– 0.18	0.15	0.27
SampEn	7.57	14.10	0.65	0.17	0.48	0.04	0.24	– 0.44	0.51	0.31
DFA α	7.76	13.95	0.55	0.10	0.27	– 0.02	0.14	– 0.29	0.25	0.34
DFA α1	9.60	19.88	0.55	0.14	0.39	– 0.05	0.20	– 0.44	0.34	0.13
DFA α2	8.78	16.40	0.57	0.11	0.31	– 0.01	0.16	– 0.32	0.30	0.67
CI-8	6.08	9.93	0.43	1.37	3.78	0.15	1.93	– 3.64	3.93	0.61
SD2	18.13	42.61	0.33	17.43	48.30	1.31	24.64	– 46.99	49.61	0.74

*RMSSD* Root mean square of successive differences of normal RR intervals; *SDNN* standard deviation of normal RR intervals; *LF* absolute power in low frequency band; *HF* absolute power in high frequency band; *ApEn* approximate entropy; *SampEn* sample entropy; *DFA* detrended fluctuation analysis; *CI-8* complexity index under 8 scales; *SD2* standard deviation of points along the line of identity of the Poincare plot; *CV* coefficient of variation; *ICC* intraclass correlation coefficient; *MDC* minimal detectable change; *LOA* limits of agreement

**Table 3:** Younger group day-to-day reliability of RR interval complexity and variability metrics

	Between Day CV (%)	Between Participant CV (%)	ICC2,1	SEM	MDC	Bias	SD bias	Lower 95% LOA	Upper 95% LOA	*P*
HR (bpm)	6.23	13.92	0.67	4.97	13.77	1.14	7.03	– 12.63	14.91	0.46
RRi (s)	6.22	13.75	0.71	0.08	0.21	– 0.01	0.11	– 0.22	0.20	0.69
RMSSD (ms)	17.88	46.46	0.81	15.48	42.91	– 3.44	21.89	– 46.35	39.47	0.47
LnRMSSD	4.42	11.29	0.79	0.22	0.60	– 0.03	0.31	– 0.63	0.58	0.70
SDNN (ms)	18.96	39.05	0.64	24.18	67.02	– 0.73	34.19	– 67.75	66.29	0.92
LnSDNN	4.34	8.08	0.59	0.24	0.67	0.03	0.34	– 0.64	0.70	0.68
LF (ms^2^)	30.72	72.82	0.56	1186.69	3289.34	– 370.70	1678.23	– 3660.04	2918.64	0.31
HF (ms^2^)	36.48	91.22	0.75	1015.31	2814.30	– 230.05	1435.87	– 3044.35	2584.25	0.46
LF (Ln)	4.43	9.68	0.72	0.41	1.13	– 0.03	0.58	– 1.16	1.11	0.83
HF (Ln)	5.38	13.38	0.78	0.45	1.24	– 0.04	0.63	– 1.28	1.20	0.77
LF/HF (ratio)	24.58	71.85	0.80	0.54	1.50	0.04	0.77	– 1.46	1.54	0.80
ApEn	3.52	5.33	0.37	0.07	0.18	– 0.003	0.09	– 0.18	0.18	0.87
SampEn	7.65	12.74	0.49	0.20	0.55	– 0.10	0.28	– 0.64	0.45	0.11
DFA α	6.42	16.69	0.84	0.06	0.18	– 0.02	0.09	– 0.20	0.16	0.35
DFA α1	6.52	22.86	0.93	0.08	0.21	– 0.005	0.11	– 0.21	0.21	0.88
DFA α2	8.98	17.68	0.69	0.10	0.26	– 0.04	0.13	– 0.30	0.22	0.17
CI-8	7.48	13.56	0.69	1.59	4.41	– 0.82	2.25	– 5.22	3.59	0.10
SD2	20.42	64.69	0.44	60.00	166.32	– 10.77	84.86	– 177.09	155.54	0.45

*RMSSD* Root mean square of successive differences of normal RR intervals; *SDNN* standard deviation of normal RR intervals; *LF* absolute power in low frequency band; *HF* absolute power in high frequency band; *ApEn* approximate entropy; *SampEn* sample entropy; *DFA* detrended fluctuation analysis; *CI-8* complexity index under 8 scales; *SD2* standard deviation of points along the line of identity of the Poincare plot; *CV* coefficient of variation; *ICC* intraclass correlation coefficient; *MDC*  minimal detectable change; *LOA* limits of agreement

### Effect of age, sex and $\dot{V}{\text{O}}_{{{\text{2peak}}}}$ on heart rate complexity

**Fig. 2:**
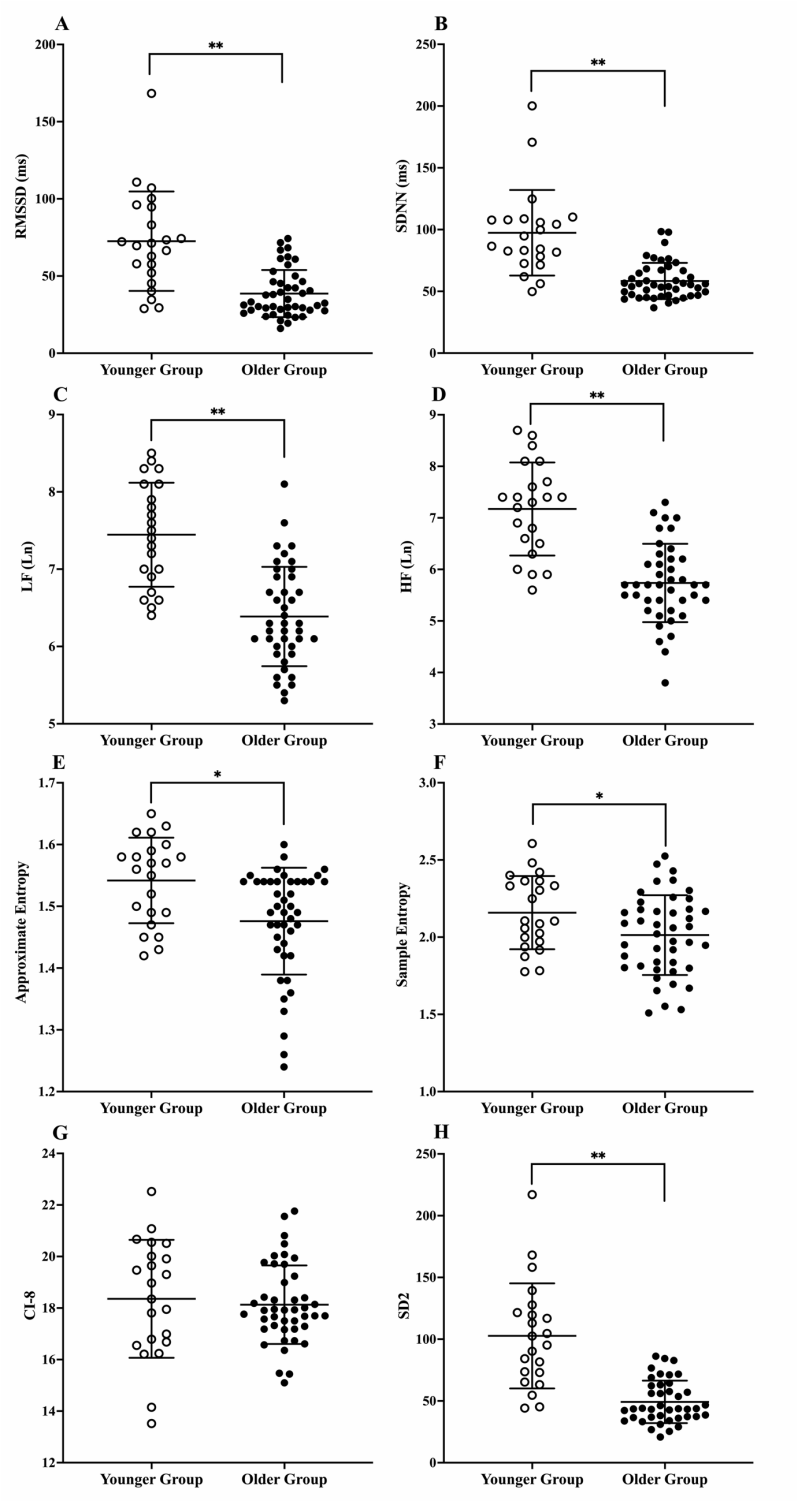
Comparisons between the younger and older groups complexity and variability metrics **A** root mean square of successive differences between normal RR intervals; **B** standard deviation of normal RR intervals; **C** low frequency power; **D** high frequency power; **E** approximate entropy; **(F)** sample entropy; **G** complexity index under 8 scales; **H** standard deviation of points along the line of identity of the Poincare plot (*P < 0.05; **P < 0.001; Data points are the mean of both days for each individual participant)

There was a significant reduction in the ApEn (*P* < 0.001; Fig. 2E), SampEn (*P* = 0.031; Fig. 2F) and SD2 (*P* < 0.001; Fig. 2H) metrics with ageing (Table 5). There was no significant effect of age on the CI-8 (*P* = 0.493; Fig. 2G; Table 5).

**Fig. 3:**
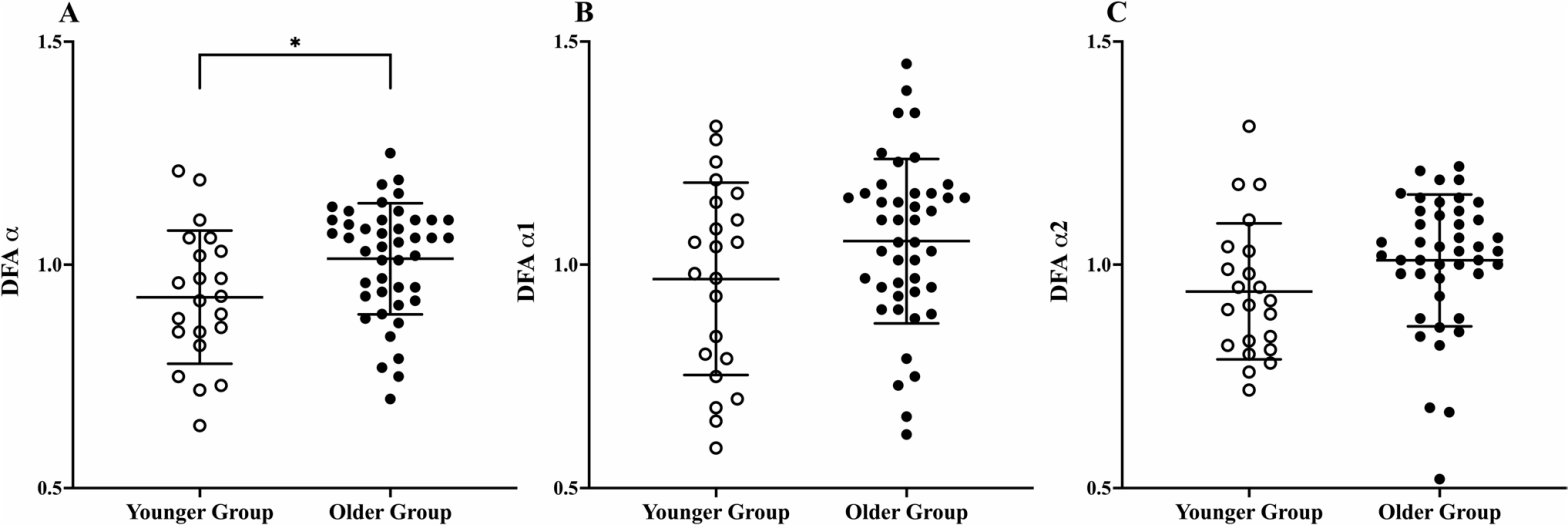
Comparisons between the younger and older groups detrended fluctuation analysis metrics **A** DFA α (box sizes 4 to $\le$ 64 data points); **B** DFA α1 (box sizes of 4 $\le$ n $\le$ 16 data points); **C** DFA α2 (box sizes of 16 ≤ n $\le$ 64 data points; *P < 0.05; Data points are the mean of both days for each individual participant)

There was no significant effect of age on the DFA α1 (*P* = 0.107; Fig. 3B) and DFA α2 (*P* = 0.147; Fig. 3C) metrics (Table 5). The DFA α metric was significantly increased with ageing (*P* = 0.029; Fig. 3A).

There was a significant effect of sex (*P* = 0.028), but not or $\dot{V}{\text{O}}_{{{\text{2peak}}}}$ (*P* = 0.822) on DFA α1, with females presenting with lower values. There was no significant effect of sex or $\dot{V}{\text{O}}_{{{\text{2peak}}}}$ on the ApEn, SampEn, DFA α, DFA α2, CI-8 and SD2 metrics (*P* > 0.05; Table 5).

### Effect of age, sex and $\dot{V}{\text{O}}_{{{\text{2peak}}}}$ on heart rate variability

There was a significant reduction in RMSSD (*P* < 0.001; Fig. 2A), SDNN (*P* < 0.001; Fig. 2B), LF power (*P* < 0.001; Fig. 2C) and HF power (*P* < 0.001; Fig. 2D) metrics with ageing (Table 5).

There was no significant effect of sex or $\dot{V}{\text{O}}_{{{\text{2peak}}}}$ on all linear HRV metrics (*P* > 0.05; Table 5).

## Discussion

### Reliability of heart rate complexity and variability metrics

The current study provides new inter-day reliability data for a range of widely utilised time-domain, frequency-domain and nonlinear HRV metrics in healthy highly active younger and older adults. The primary findings of this investigation reveal all linear HRV metrics in both the younger adult and older adult groups to exhibit moderate to good inter-day reliability, as indicated by ICCs ranging from 0.56 to 0.88 (Tables 2 and 3). Similarly, the majority of nonlinear HRV metrics demonstrated moderate to excellent inter-day reliability with ICCs ranging from 0.55 to 0.93 (Tables 2 and 3). There were exceptions however, with ApEn, SampEn and SD2 metrics of the YG, and the SD2 metric of the OG exhibiting poor relative reliability, as shown by ICCs of less than 0.50 (Tables 2 and 3). This variability in the inter-day reliability of HRV metrics can likely be attributed to the sensitivity of the ANS and the influence of various individual internal and external factors that can be challenging to control (Fatisson et al. 2016).

It has been suggested that the assessment of test–retest reliability should not rely solely on ICCs (Weir 2005). This viewpoint is supported by the current study, with the ApEn, SampEn and CI-8 HRV metrics displaying ICCs ranging from 0.37 to 0.69, indicating poor to moderate relative reliability (Tables 2 and 3). However, these metrics exhibited low SEM values (ranging from 0.06 to 0.20) and low between day CVs (ranging from 2.95% to 7.65%), which suggests high absolute retest reliability. This apparent contradiction can be explained by the homogeneous population recruited and low between participant CVs for these specific metrics, leading to low relative but high absolute reliability (Atkinson and Nevill 1998; Weir 2005). In contrast, the SD2 metric showed both low relative reliability (ICCs ranging from 0.33 to 0.44) and low absolute reliability (between day CVs of 18.13% to 20.42% and SEM values of 17.43 to 60.00). Similarly, the frequency-domain metrics LF, HF, and LF/HF also exhibited low absolute reliability (Tables 2 and 3). These findings indicate that specific HRV metrics may present significant challenges when used to detect intervention/treatment effects or individual changes over time. Consequently, the HRV metrics with low relative and absolute reliability may not be suitable in specific research contexts, especially those with limited sample sizes or small intervention/treatment effects.

ICCs and SEM of the SampEn and DFA metrics for both age groups in the current study are comparable to those reported by Maestri et al. (2007a) who examined HRV inter-day reliability in healthy adults with a mean age of 38 years (range 26 to 56 years). Accordingly, the LnRMSSD, LnSDNN, LnLF, and LnHF metrics of both age groups produced similar ICCs to those reported for healthy young students aged between 18 and 39 years (Uhlig et al. 2020), in addition to comparable between day CVs and SEM to healthy trained young adults (aged 21.5 ± 1.4 years; Al Haddad et al. 2011). The corroboration between reliability studies improves confidence in the expected retest error of HRV metrics. However, it also emphasises the high level of variance in certain HRV metrics (i.e., LF, HF, LF/HF and SD2), as well as the difficulty facing researchers in sufficiently powering studies which are utilising HRV measurements across multiple visits and/or during longitudinal studies.

The study builds upon previous HRV reliability research providing inter-day reliability data for short-term resting HRV measurements for younger and importantly older adults across a range of widely utilised HRV metrics. The reliability data in Tables 2 and 3 provides a resource for researchers to reference when calculating sample sizes for future HRV studies with healthy adult participants. Importantly, given the disparity in the reliability of different HRV metrics (ICCs, 0.33 to 0.93; Between day CVs, 2.9 to 36.5; Tables 2 and 3), study sample size is recommended to be based upon the chosen metric with the lowest reliability to reduce the likelihood of a type I or type II error across all metrics. In addition, the reliability statistics also allow for the assessment of whether there is a true intervention effect or individual change in HRV metrics within a study and not just a result of biological and measurement error.

### Effect of age, sex and $\dot{V}{\text{O}}_{{{\text{2peak}}}}$ on heart rate complexity and variability

The current study findings demonstrate a significant age-related decline in linear (RMSSD, LnRMSSD, SDNN, LnSDNN, LF, HF) and nonlinear (ApEn, SampEn and SD2) HRV metrics (Tables 4 and 5; Fig. 2), corroborating the findings of a broad body of literature which has assessed the effect of age on heart rate complexity and variability (Kaplan et al. 1991; Iyengar et al. 1996; Jensen-Urstad et al. 1997; Umetani et al. 1998; Pikkujamsa et al. 1999; Beckers et al. 2006; Goff et al. 2010; Voss et al. 2015; Hernandez-Vicente et al. 2020). An age-related decrease in both the linear and nonlinear HRV metrics is expected, primarily driven by alterations in the ANS, characterised by a decline in parasympathetic activity and an increase in sympathetic drive (Seals and Esler 2000).

**Table 4:** Mean HRV metrics for age groups and effect size comparisons

	YG *N* = 22	OG *N* = 44	Hedges’ *g*	Hedges’ g lower 95% CI	Hedges’ g upper 95% CI
HR (bpm)	61.75 ± 75	54.24 ± 6.28	1.12	0.57	1.66
RRi (s)	1.00 ± 0.14	1.13 ± 0.13	1.03	0.49	1.57
RMSSD (ms)	72.56 ± 33.64	38.60 ± 16.95	1.51	0.93	2.08
LnRMSSD	4.18 ± 0.45	3.56 ± 0.38	1.52	0.95	2.10
SDNN (ms)	97.40 ± 38.16	58.40 ± 16.33	1.66	1.08	2.25
LnSDNN	4.51 ± 0.33	4.03 ± 0.24	1.77	1.17	2.36
LF (ms^2^)	2197.61 ± 1445.90	763.81 ± 582.24	1.48	0.91	2.05
HF (ms^2^)	1958.89 ± 1692.68	431.53 ± 345.17	1.49	0.92	2.07
LF (Ln)	7.45 ± 0.67	6.39 ± 0.64	1.62	1.04	2.20
HF (Ln)	7.17 ± 0.91	5.73 ± 0.76	1.75	1.16	2.34
LF/HF ratio	1.71 ± 1.16	2.71 ± 2.97	− 0.39	− 0.91	0.12
ApEn	1.54 ± 0.08	1.48 ± 0.10	0.82	0.29	1.35
SampEn	2.16 ± 0.28	2.01 ± 0.28	0.57	0.05	1.09
DFA α	0.93 ± 0.15	1.01 ± 0.14	− 0.64	− 1.16	− 0.12
DFA α1	0.97 ± 0.22	1.05 ± 0.21	− 0.43	− 0.95	0.09
DFA α2	0.94 ± 0.17	1.01 ± 0.17	− 0.46	− 0.98	0.05
CI-8	18.36 ± 2.25	18.13 ± 1.52	0.12	− 0.39	0.64
SD2	110.84 ± 29.67	49.21 ± 10.08	1.87	1.27	2.48

*YG* Younger group; *OG* older group; *HR* heart rate; *RRi* time between two successive R-waves of an ECG; *RMSSD* root mean square of successive differences between normal RR intervals; *SDNN* standard deviation of normal RR intervals; *LF* absolute power in low frequency band; *HF* absolute power in high frequency band; *ApEn* approximate entropy; *SampEn* sample entropy; *DFA* detrended fluctuation analysis; *CI-8* complexity index under 8 scales; *SD2* standard deviation of points along the line of identity of the Poincare plot; data are mean ± SD of both days measurements

**Table 5:** Multiple linear regression model statistics

	Overall regression model			Age (years)		Sex (M/F) [F]		$\dot{V}{\text{O}}_{{{\text{2peak}}}}$ (L min^−1^)	
	Adjusted *R*^2^	*F* (6, 59)	*P*	*Β* [95% CI]	*t* *P*	*Β* [95% CI]	*t* *P*	*Β* [95% CI]	*t* *P*
HR (bpm)	0.267	8.884	** < 0.001**	– 0.236 [– 0.335, – 0.138]	4.800 ** < 0.001**	– 0.091 [– 5.518, 5.336]	0.034 0.973	– 2.121 [– 4.896, 0.653]	1.528 0.135
RRi (s)	0.197	6.305	** < 0.001**	0.003 [0.001, 0.005]	4.033 ** < 0.001**	– 0.002 [– 0.108, 0.103]	0.044 0.965	0.032 [– 0.002, 0.086]	1.167 0.248
RMSSD (ms)	0.362	13.270	** < 0.001**	– 0.853 [– 1.188, – 0.519]	5.097 ** < 0.001**	15.550 [– 2.915, 34.010]	1.683 0.097	2.107 [– 7.332, 11.550]	0.446 0.657
LnRMSSD	0.347	12.520	** < 0.001**	– 0.015 [– 0.021, – 0.009]	4.980 ** < 0.001**	0.255 [– 0.083, 0.592]	1.509 0.136	0.051 [– 0.121, 0.223]	0.592 0.556
SDNN (ms)	0.379	14.230	** < 0.001**	– 1.019 [– 1.375, – 0.662]	5.721 ** < 0.001**	1.126 [– 18.520, 20.770]	0.115 0.909	1.736 [– 8.309, 11.780]	0.345 0.731
LnSDNN	0.412	16.190	** < 0.001**	– 0.012 [– 0.017, – 0.008]	5.986 ** < 0.001**	0.022 [– 0.206, 0.250]	0.196 0.846	0.035 [– 0.081, 0.152]	0.603 0.549
LF (ms^2^)	0.303	9.998	** < 0.001**	– 35.730 [– 51.200, – 20.270]	4.624 ** < 0.001**	– 7.820 [– 862.100, 846.500]	0.018 0.985	114.200 [– 328.400, 556.800]	0.517 0.608
HF (ms^2^)	0.353	12.280	** < 0.001**	– 41.560 [– 57.410, – 25.700]	5.246 ** < 0.001**	404.900 [– 470.800, 1281.000]	0.925 0.359	– 75.550 [– 529.200, 378.100]	0.333 0.740
LF (Ln)	0.367	13.000	** < 0.001**	– 0.026 [– 0.036, – 0.016]	5.129 ** < 0.001**	– 0.158 [– 0.724, 0.407]	0.559 0.578	0.099 [– 0.194, 0.392]	0.675 0.502
HF (Ln)	0.431	16.620	** < 0.001**	– 0.037 [– 0.049, – 0.024]	5.913 ** < 0.001**	0.449 [– 0.243, 1.143]	1.298 0.199	0.052 [– 0.307, 0.411]	0.289 0.773
LF/HF ratio	0.064	2.425	0.075	0.023 [– 0.016, 0.061]	1.187 0.239	– 1.921 [– 4.035, 0.192]	1.819 0.074	– 0.442 [– 1.537, 0.654	0.807 0.423
ApEn	0.202	6.478	** < 0.001**	– 0.002 [– 0.003, – 0.001]	3.917 ** < 0.001**	0.008 [– 0.057, 0.073]	0.243 0.809	– 0.023 [– 0.056, 0.012]	1.366 0.177
SampEn	0.122	4.003	**0.001**	– 0.004 [– 0.008, – 0.0004]	2.203 **0.031**	0.132 [– 0.073, 0.337]	1.291 0.202	– 0.032 [– 0.137, 0.073]	0.611 0.544
DFA α	0.142	4.573	**0.005**	0.002 [0.0002, 0.004]	2.242 **0.029**	– 0.079 [– 0.187, 0.029]	1.469 0.147	0.017 [– 0.038, 0.072]	0.621 0.537
DFA α1	0.167	5.350	**0.002**	0.002 [– 0.0005, 0.005]	1.635 0.107	– 0.171 [– 0.323, – 0.019]	2.245 **0.028**	0.009 [– 0.069, 0.086]	0.226 0.822
DFA α2	0.020	1.443	0.239	0.002 [– 0.0006, 0.004]	1.469 0.147	– 0.035 [– 0.162, 0.091]	0.557 0.579	0.018 [– 0.047, 0.082]	0.551 0.584
CI-8	– 0.026	0.457	0.714	– 0.009 [– 0.038, 0.018]	0.689 0.493	– 0.207 [– 1.746, 1.332]	0.269 0.788	– 0.369 [– 1.156, 0.418]	0.937 0.352
SD2	0.482	20.230	** < 0.001**	– 1.501 [– 1.933, – 1.070]	6.969 ** < 0.001**	8.167 [– 15.650, 31.980]	0.686 0.495	– 8.328 [– 20.670, 4.012]	1.350 0.182

	Age × sex		Age × $\dot{V}{\text{O}}_{{{\text{2peak}}}}$		Sex × $\dot{V}{\text{O}}_{{{\text{2peak}}}}$	
	β [95% CI]	*t* *P*	β [95% CI]	*t* *P*	β [95% CI]	*t* *P*
HR (bpm)	0.144 [– 0.1564, 0.445]	0.961 0.341	0.116 [– 0.039, 0.271]	1.489 0.142	– 6.782 [– 15.070, 1.507]	1.637 0.107
RRi (s)	– 0.003 [– 0.009, 0.003]	0.993 0.325	– 0.002 [– 0.005, 0.001]	1.123 0.266	0.123 [– 0.039, 0.286]	1.513 0.136
RMSSD (ms)	– 0.998 [– 1.989, – 0.007]	2.015 **0.049**	– 0.190 [– 0.703, 0.323	0.742 0.461	16.900 [– 10.410, 44.210]	1.238 0.221
LnRMSSD	– 0.013 [– 0.032, 0.006]	1.364 0.178	– 0.003 [– 0.012, 0.007]	0.528 0.599	0.173 [– 0.349, 0.696]	0.662 0.511
SDNN (ms)	– 0.026 [– 1.138, 1.085]	0.048 0.962	– 0.094 [– 0.669, 0.481]	0.327 0.745	26.260 [– 4.367, 56.890]	1.716 0.092
LnSDNN	< 0.001 [– 0.013, 0.013]	0.030 0.976	< – 0.001 [– 0.007, 0.006]	0.155 0.877	0.280 [– 0.077, 0.637]	1.568 0.122
LF (ms^2^)	– 14.900 [– 61.150, 31.360]	0.645 0.522	– 15.950 [– 40.280, 8.387]	1.313 0.194	1273.000 [– 3.202, 2548.000]	1.998 0.050
HF (ms^2^)	– 34.490 [– 82.220, 13.250]	1.447 0.153	– 3.728 [– 28.840, 21.380]	0.2970 0.767	551.800 [– 764.800, 1869.000]	0.839 0.405
LF (Ln)	– 0.003 [– 0.034, 0.028]	0.178 0.859	– 0.004 [– 0.021, 0.012]	0.536 0.594	0.925 [0.074, 1.775]	**2.117** **0.034**
HF (Ln)	– 0.020 [– 0.059, 0.018]	1.054 0.297	– 0.002 [– 0.022, 0.018]	0.201 0.841	0.267 [– 0.801, 1.335]	0.501 0.618
LF/HF ratio	– 0.025 [– 0.145, 0.094]	0.421 0.676	– 0.029 [– 0.092, 0.034]	0.925 0.359	1.150 [– 2.144, 4.443]	0.699 0.487
ApEn	0.002 [– 0.001, 0.006]	1.259 0.213	0.001 [– 0.001, 0.003]	1.302 0.198	– 0.065 [– 0.166, 0.035]	1.300 0.199
SampEn	– 0.007 [– 0.019, 0.004]	1.262 0.212	– 0.001 [– 0.007, 0.005]	0.353 0.726	0.070 [– 0.249, 0.389]	0.439 0.662
DFA α	0.005 [– 0.001, 0.113]	1.724 0.089	0.001 [– 0.002, 0.004]	0.629 0.532	0.006 [– 0.161, 0.173]	0.072 0.943
DFA α1	0.009 [0.001, 0.018]	2.244 **0.029**	0.001 [– 0.004, 0.005]	0.289 0.773	0.129 [– 0.098, 0.357]	1.135 0.261
DFA α2	0.001 [– 0.006, 0.009]	0.389 0.698	0.001 [– 0.003, 0.005]	0.514 0.609	– 0.097 [– 0.297, 0.103]	0.971 0.336
CI-8	– 0.033 [– 0.120, 0.054]	0.769 0.445	– 0.008 [– 0.053, 0.037]	0.337 0.738	1.458 [– 0.939, 3.856]	1.217 0.229
SD2	– 1.281 [– 2.577, 0.016]	1.979 0.053	– 0.148 [– 0.830, 0.534]	0.436 0.665	– 9.758 [– 45.520, 26.000]	0.547 0.587

Significant *P *values are denoted in bold

$\dot{V}{\text{O}}_{{{\text{2peak}}}}$ Peak oxygen uptake; *HR* heart rate; *RRi* time between two successive R-waves of an ECG; *RMSSD* root mean square of successive differences between normal RR intervals; *SDNN* standard deviation of normal RR intervals; *LF* absolute power in low frequency band; *HF* absolute power in high frequency band; *ApEn* approximate entropy; *SampEn* sample entropy; *DFA* detrended fluctuation analysis; *CI-8* complexity index under 8 scales; *SD2* standard deviation of points along the line of identity of the Poincare plot; data are mean ± SD of both days measurements

Despite age-related differences in all other HRV metrics, there was no significant effect of age on the nonlinear DFA α1 and α2 metrics (Table 5; Fig. 3B and C). Mean DFA α1 and α2 values were close to 1.0 (i.e., 1/f or pink noise), indicative of a healthy physiological signal of high complexity that is exhibiting both short and long-range fractal-like correlations (Peng et al. 1995). These findings are comparable to previous research which also found no age-related difference in the DFA α1 and α2 metric (Vuksanovic and Gal 2005; Schmitt and Ivanov 2007; Wiersema et al. 2022). Seminal research exploring the effect of age on the fractal behaviour of RR interval time series observed healthy older adults (α2 = 0.75 ± 0.17) to have a significant decline in long-range fractal correlations, in comparison to healthy younger adults (α2 = 0.99 ± 0.10; Iyengar et al. 1996). The mean age of the older group in the study of Iyengar et al. (1996) was greater than the older group of the current study (74 years vs 59 years), which may partly explain the difference in findings between the studies, as well as the high activity levels of the older participants of the current study. It is important to note that despite recruiting a homogenous sample, several participants did produce α1 and α2 values closer to 0.5 and 1.5 (Fig. 3B and C). Such between participant variation is expected, occurring to differing extents for all HRV metrics (Tables 2 and 3) and highlights the importance of also accounting for the inter-individual variability of HRV metrics when seeking to understand the utility of HRV in different populations.

The findings of the current study demonstrate no significant age-related change in the nonlinear CI-8 metric (Fig. 2G; Table 5). Like the DFA α1 and α2 metrics, the CI-8 metric captures the structural and dynamical behaviour of the RR interval time series over multiple scales (Costa et al. 2002). Accordingly, the complexity (DFA and CI-8) of the study participants’ RR interval time series is suggestive of their cardiovascular systems ability to adapt to physiologic perturbations and respond quickly to challenges to maintaining homeostasis (Peng et al. 2009; Manor and Lipsitz 2013). The mixed findings of the effect of age on different HRV metrics highlights the necessity of employing multiple heart rate complexity and variability metrics when analysing RR interval times series. If only specific time-domain, frequency-domain or non-linear HRV metrics are utilised, studies may fail to capture different linear and nonlinear aspects of the signal, therefore potentially missing important information on cardiac interval dynamics. However, the choice and combination of HRV metrics by researchers is also likely to be dependent on the research context; with different HRV metrics better suited to capturing specific properties and/or changes in cardiac interval dynamics, in addition to the redundancy of combining HRV metrics which measure similar HRV properties (Maestri et al. 2007b).

The current study included male (*N* = 50) and female (*N* = 16) participants. Sex differences in HRV are well documented and are influenced by physiological, hormonal, and neural factors (Koenig and Thayer 2016). Moreover, sex-related differences in HRV may be more pronounced in younger adults, when compared to older adults (Maria et al. 2023). It should be noted that the current study did not control for menstrual cycle phase or hormone changes due to the menopause, which are known to effect HRV (Aubert et al. 2003; Maria et al. 2023). Sex did not significantly predict the HRV metrics in the current study, except for the DFA α1 metric (Table 5). The significant effect of sex indicates that females present with lower α1 value in comparison to males. Such differences in α1 is suggestive of a notable change in the short-range fractal correlation properties of HRV and an alteration in sympathetic and vagal activation (Tulppo et al. 2005).

While sex was not significantly predictive of the HRV metrics, the beta coefficients indicate a trend towards females having higher values in HRV metrics primarily associated with parasympathetic activity (i.e., HF power and RMSSD) in comparison to males. There is evidence to support an increase in parasympathetic modulation (as indicated by absolute HF power) in females compared to males (Koenig and Thayer 2016). However, evidence is argued to be inconclusive with heterogeneity in study findings, likely emanating from differences in study methodology and analysis methods (Maria et al. 2023).

Aerobic physical activity has been shown to have positive effects on measures of HRV in both younger and older adults, when compared to sedentary age matched individuals, through enhanced autonomic balance, improved baroreflex sensitivity and cardiac adaptations (Aubert et al. 2003). To capture the effect of inherent biological ageing on HRV (i.e., individuals unaffected by sedentary behaviour or underlying pathologies) all participants of the current study were recruited to be in full health and regular exercisers closely matched for physical activity levels and aerobic fitness (Table 1). Although the YG did present with a higher absolute aerobic fitness as measured by $\dot{V}{\text{O}}_{{{\text{2peak}}}}$ (YG $\dot{V}{\text{O}}_{{{\text{2peak}}}}$ = 3.5 ± 1.0 L min^−1^ vs. OG $\dot{V}{\text{O}}_{{{\text{2peak}}}}$ = 3.0 ± 0.8 L min^−1^), $\dot{V}{\text{O}}_{{{\text{2peak}}}}$ was not significantly predictive of any HRV metric (Table 5).

### Limitations

The current study only assessed the reliability of HRV metrics derived from short-term RR interval measurements in healthy active younger and older adults during free-breathing wakeful supine rest. Due to the sensitivity of the ANS to various external and internal factors (Fatisson et al. 2016), caution is advised when extrapolating the reliability data reported herein to HRV metrics derived from RR interval measurements performed under different conditions. The current study was limited to the assessment of inter-day reliability and did not assess the intra-day reliability of the HRV metrics. Given the sensitivity of the ANS, it is probable the inter-day variation in HRV largely reflects biological error, whereas intra-day variation in HRV would likely provide a closer insight into the measurement error.

The current study assessed a range of time-domain, frequency-domain and nonlinear HRV metrics, which are extensively studied and widely accepted to provide valuable information regarding ANS function in ageing, between sexes and in athletes (Koenig and Thayer 2016; Shaffer and Ginsberg 2017; Lundstrom et al. 2023). However, it is important to highlight that the study does not provide a comprehensive list of available HRV metrics. Notably, the study did not include HRV metrics from the major families of symbolic dynamics, predictability, and empirical mode decomposition (Maestri et al. 2007b). Researchers should specifically consider using the symbolic dynamic metric, one variation pattern (1VP) and empirical mode decomposition metric, IMAI2. The IVP and IMAI2 metrics have been shown to provide additive predictive value independent to clinical predictors when assessing chronic heart failure patients (Maestri et al. 2007b) and detect experimentally induced changes in autonomic cardiovascular regulation in healthy individuals (Guzzetti et al. 2005).

The nonlinear HRV metric, ApEn, was included in the current study as a metric from the entropy family, which can assess the irregularity or randomness of an RR interval time series (Pincus 1991). However, the calculation of ApEn presents notable limitations due to its self-matching that may affect its interpretation (Richman and Moorman 2000). ApEn exhibits sensitivity to data length, particularly in cases of short data sequences such as RR interval time series, leading to potentially biased results due to its reliance on pattern identification within the arbitrarily specified tolerance parameter, “*r*”. Moreover, ApEn’s susceptibility to self-matching can cause relative inconsistencies; meaning if the ApEn of a time series is higher than another time series, it should remain higher under all conditions, however, it does not always remain higher (Richman and Moorman 2000). Despite ApEn demonstrating high absolute retest reliability, researchers are advised to account for these limitations when using ApEn for HRV analysis.

## Conclusion

The current findings show that widely used HRV metrics derived from short-term (30-min) RR interval measurements are reproducible between days in healthy, highly active younger and older adults. However, there is a disparity in the inter-day reliability of different HRV metrics, with certain metrics presenting with a higher level of variance (i.e., LF, HF, LF/HF and SD2). Both linear and nonlinear HRV metrics capture different aspects of cardiac interval dynamics; therefore, researchers should not exclude metrics based solely on their reliability. Instead, studies should be designed appropriately based upon the chosen HRV metrics to increase the probability of detecting a true effect. This study also extends upon previous research by demonstrating a significant age-related decline in the majority of linear and nonlinear HRV metrics assessed. However, the participants’ sex and $\dot{V}{\text{O}}_{{{\text{2peak}}}}$ did not significantly influence the HRV metrics.

## Data Availability

Data analysis software application used (SPSS and MATLAB) openly available.

## Abbreviations

ANOVA: Analysis of variance
ANS: Autonomic nervous system
ApEn: Approximate entropy
CI-8: Complexity index under 8 scales
CV: Coefficient of variation
DFA: Detrended fluctuation analysis
HF: High frequency power
HRV: Heart rate variability
ICC2,1: Intraclass correlation coefficient
IET: Incremental exercise test
LF: Low frequency power
LOA: Limits of agreement
MDC: Minimal detectable change
MSE: Multiscale entropy
OG: Older group
RMSSD: Root mean square of successive differences between normal RR intervals
SampEn: Sample entropy
SDNN: Standard deviation of normal RR intervals
SD2: Standard deviation of points along the line of identity of the Poincare plot
SEM: Standard error of measurement
$\dot{V}{\text{O}}_{{{\text{2peak}}}}$: Peak oxygen uptake
$\dot{V}{\text{E}}/\dot{V}{\text{O}}_{{2}}$: Ventilatory equivalent of oxygen
$\dot{V}{\text{E}}/\dot{V}{\text{CO}}_{{2}}$: Ventilatory equivalent of carbon dioxide
YG: Younger group
